# The Role of p53 in Regulating Chronic Inflammation and PANoptosis in Diabetic Wounds

**DOI:** 10.14336/AD.2024.0212

**Published:** 2024-02-12

**Authors:** Wenjie He, Ming Tang, Rifang Gu, Xingqian Wu, Xinrui Mu, Xuqiang Nie

**Affiliations:** ^1^College of Pharmacy, Zunyi Medical University, Zunyi 563006, China.; ^2^Key Lab of the Basic Pharmacology of the Ministry of Education & Joint International Research Laboratory of Ethnomedicine of Ministry of Education, Zunyi Medical University, Zunyi 563006, China.; ^3^Department of Structural Biology, St. Jude Children’s Research Hospital, Memphis 38105, USA.; ^4^School Medical Office, Zunyi Medical University, Zunyi 563006, China.

**Keywords:** diabetic wounds, p53, inflammation, senescence, PANoptosis, ferroptosis, regulatory factors

## Abstract

Diabetic wounds represent a formidable challenge in the clinical management of diabetes mellitus, markedly diminishing the patient's quality of life. These wounds arise from a multifaceted etiology, with the pathophysiological underpinnings remaining elusive and complex. Diabetes precipitates neuropathies and vasculopathies in the lower extremities, culminating in infections, ulcerations, and extensive tissue damage. The hallmarks of non-healing diabetic wounds include senescence, persistent inflammation, heightened apoptosis, and attenuated cellular proliferation. The *TP53* gene, a pivotal tumor suppressor frequently silenced in human malignancies, orchestrates cellular proliferation, senescence, DNA repair, and apoptosis. While p53 is integral in cell cycle regulation, its role in initial tissue repair appears to be deleterious. In typical cutaneous wounds, p53 levels transiently dip, swiftly reverting to baseline. Yet in diabetic wounds, protracted p53 activation impedes healing via two distinct pathways: i) activating the p53-p21-Retinoblastoma (RB) axis, which halts the cell cycle, and ii) upregulating the cGAS-STING and nuclear factor-kappaB (NF-κB) cascades, instigating ferroptosis and pyroptosis. Furthermore, p53 intersects with various metabolic pathways, including glycolysis, gluconeogenesis, oxidative phosphorylation, and autophagy. In diabetic wounds, p53 may drive metabolic reprogramming, thus potentially derailing macrophage polarization. This review synthesizes case studies investigating the therapeutic modulation of p53 in diabetic wounds care. In summation, p53 modulates chronic inflammation and cellular aging within diabetic cutaneous wounds and is implicated in a novel cell death modality, encompassing ferroptosis and pyroptosis, which hinders the reparative process.

## 1. Introduction

In individuals with diabetes mellitus, impaired wound healing is a critical issue that can culminate in the formation of chronic non-healing ulcers, especially diabetic foot ulcers (DFU). These lesions represent a severe and potentially life-threatening complication that may necessitate amputation, thereby exerting substantial economic, social, and psychological burdens [1, 2]. Cellular senescence has been recognized as a pivotal impediment to the healing of diabetic wounds (DW) [3-6]. Progressive upregulation of senescence and apoptosis markers such as p53- Cdkn1a (p21) -p16, β-galactosidase (SA-β-gal), and B-cell lymphoma-2 (Bcl-2)-associated X protein (Bax), accompanied by diminished bcl-2- expression, has been documented in these wounds [7-10]. Inhibition of p53 activity has been shown to expedite wound repair processes [11-13].

The *TP53* tumor suppressor gene, a guardian of the genome located on chromosome 17p13, orchestrates cell cycle regulation, apoptosis, and cellular proliferation. It induces p21 expression, which subsequently binds to cyclin complexes, inhibiting cyclin-dependent kinase activity and safeguarding the genome during cell division [14]. The p53-p21 axis serves as a protective mechanism against aberrant cellular proliferation by regulating cyclin E- cyclin-dependent kinase 2 (CDK2) activity [15]. In certain senescent pathologies, hyperactivation of p53 and p21 results in cell cycle arrest at the G1-S or G2-M transition and reduced cellular proliferation [16, 17]. Originally described by Hayflick in 1961, cellular senescence denotes a state of irreversible growth arrest under stressors such as DNA damage, radiation, or telomere attrition [18]. Markers like p53 and p21 are instrumental in identifying senescence *in vivo* and can also reflect biological aging when detected in peripheral blood [19, 20]. Interestingly, while p53 is known to induce senescence, this function appears to be differentiated from its role in pluripotent embryonic stem cells, where senescence and cycle arrest do not correlate with p53 activity [21]. Additionally, the p21 transcript variant 2, which increases with age, may serve as an aging biomarker [22].

p53 plays a paradoxical role in autophagy regulation: it promotes autophagy in the nucleus while suppressing it in the cytoplasm. It activates mTOR's (mammalian target of rapamycin) upstream regulators, such as damage-regulated autophagy modulators and AMP-activated protein kinase (AMPK) [23], and is implicated in Protein kinase B (AKT) pathway activation, influencing reactive oxygen species (ROS) production and the senescence phenotype. However, p53 activation is not solely responsible for inducing cell cycle arrest [24].

Emerging evidence also implicates p53 in the development of insulin resistance and adipogenesis modulation [25]. Elevated p53 in white adipose tissue promotes senescence and chronic inflammation, exacerbating systemic insulin resistance. Conversely, p53 is not essential for brown adipose tissue development but, when activated in high-fat diet-induced obese mice, it enhances thermogenesis and brown adipose marker gene expression, thereby mitigating adiposity [26].

Moreover, p53 contributes to inflammatory processes. Acetylated p53 amplifies oxidative stress and inflammatory pathways in diabetic endothelial cells, leading to endothelial dysfunction [27]. It also influences macrophage inflammation and pyroptosis in diabetes, although these effects can be reversed through p53 deacetylation [28]. Nevertheless, the role of p53 in inflammation is complex; for instance, macrophages deficient in p53 are more prone to streptozotocin-induced diabetes and exhibit elevated inflammatory cytokine production and signal transducer and activator of transcription (STAT)-1 phosphorylation [29].

The intricate involvement of p53 in DW healing is multifaceted, acting as a linchpin within a network of molecular pathways that regulate cell cycle progression, apoptosis, and cellular senescence, as well as metabolic, inflammatory, and autophagic processes. This review delves into the influence of p53 on these diverse biological responses, which are critical in the context of DW healing. Moreover, it discusses potential therapeutic strategies targeting these pathways, with a particular emphasis on sugar-based treatments, underscoring the necessity for continued research in this domain.

## 2. The cell cycle suppressor p53.

### 2.1 Regulatory factors of p53.

The p53 signaling pathway is a well-characterized cascade that exerts profound control over the cell cycle. Influenced by various signaling entities such as NF-E2-related factor 2 (Nrf2) and sirtuin 1 (SIRT1), p53 acts as a central hub for potential crosstalk among different regulatory pathways implicated in cellular senescence [30-32]. p53 is pivotal in orchestrating cell growth, DNA repair, and programmed cell death [33]. Downstream, p21, a transcriptional target of p53, serves as a cell cycle inhibitor, impeding the activity of various cyclin-CDK complexes, thus inducing cell cycle arrest in the G1-S phase and contributing to cellular senescence [34]. Furthermore, p53 induces the expression of p16, another cycle inhibitor that constrains CDK activity and arrests the cell cycle at the G0/G1 phase. Certain compounds, like black cohosh, have been reported to suppress the p53-p21 axis, thereby delaying senescence [35, 36].

Positive regulators of p53 include Caveolin-1-PTRF (polymerase I and transcript release factor), which upon upregulation due to oxidative stress, can activate p53 and lead to senescence in skin fibroblasts [8]. nucleotide-oligomerization domain-like receptor subfamily C3 (*NLRC3*) has also been identified as a promoter of p53, with its absence expediting wound healing through enhanced inflammatory and proliferative responses [37]. Inhibition of SIRT1 in keratinocytes leads to increased acetylation of p53 and a decrease in autophagy, resembling patterns observed in senescent fibroblasts [38, 39] (Fig 2). While Brahma-related gene-1 (BRG1) is known to influence colorectal cancer (CRC) cell senescence by modulating the BRG1-SIRT1-p53 axis [40], its role in skin-related processes remains unexplored.

Conversely, p53 is also subject to negative regulation. For instance, the lncRNA-H19 in diabetic skin fibroblasts downregulates p53 activity and Growth Differentiation Factor 15 (GDF15) release, thereby alleviating cell cycle arrest, enhancing macrophage infiltration, and promoting DW healing [11]. Dysregulated Phosphatase and tensin homolog (PTEN) expression, observed in diabetes and dermal fibrosis, attenuates p53 phosphorylation [41]. Proteins such as iASPP (inhibitor of apoptosis stimulating p53 protein) and *TP53*-binding protein 1 (53BP1) serve as negative regulators of p53, and their deletion or inhibition can impact keratinocyte migration and potentially decelerate wound healing [42, 43]. Moreover, miRNA-221-3p from endothelial progenitor cells (EPCs) may target the p53 pathway in diabetic skin ulcers [44], and silencing of LncRNA NORAD in aged apolipoprotein E-deficient (APOE) mice appears to activate the p53-p21 axis, enhancing cell proliferation [45].

Silencing Glutaredoxin-1 (Grx1) can also trigger the p53-p21-p16 pathway, with p16 impeding the CDK4-mediated G1-S transition, leading to G1 phase arrest and senescence [36]. The p53-p21 axis further engages the p16-RB signaling pathway; p16, part of the INK4 family, inhibits RB phosphorylation by CDK4/6-cyclinD complexes, thus blocking E2 factor (E2F) and causing G1 phase arrest and senescence. However, p53 primarily inhibits CDK1/2 activity [8, 33] (Table 1).

**Table 1:** P53 upstream and downstream regulatory factors.

Direction of regulation	Upstream factors	Pathway	Mechanism	Ref.
**Positive regulation**	Caveolin-1, PTRF	Caveolin-1-PTRF-Mdm2-p53-p21	High glucose-triggered oxidative stress leads to activation of the Caveolin-1/PTR pathway, which separates Mdm2 from p53 and activates p53, inducing premature senescence in (primary) skin fibroblasts.	[8]
	Bax-Casepase-3	p53-Bax-Casepase-3	P53 leads to an increase in apoptosis factors Bax and Casepase-3. Local silencing of p53 reduces the expression of Bax and Caspase3.	[10, 45]
	NLRC3	NLRC3-p53	NLRC3 induced ubiquitination activation of p53 in (primary) skin fibroblasts.	[37]
	SIRT1	SIRT1-p53	Silencing SIRT1 in keratinocytes and skin fibroblasts can reduce p53 deacetylation.	[38]
**Negative Regulation**	CDK1/2	p53-p21-CDK1/2	In the wound of diabetes, the up-regulated p53 inhibits the activity of CDK1/2, etc., leading to decreased cell proliferation.	[8, 33]
	lncRNA-H19	lncH19-p53-GDF15	LncH19 alleviates cell cycle arrest in primary skin fibroblasts and increases macrophage infiltration in damaged tissues by inhibiting p53 activity and GDF15 release.	[11]
	Grx1	Grx1-p53-p21	The silencing of Grx1 leads to p53 activation in 293T and U87 cells.	[36]
	BRG1	BRG1-SIRT1-p53	BRG1 can inhibit the SIRT1-p53 axis.	[40]
	PTEN	PTEN-p53	PTEN can inhibit the phosphorylation of p53 in HK-1 cells.	[41]
	iASPP-AHNAK	iASPP-AHNAK-p53	The absence of iASPP and 53BP1 can lead to impaired migration of keratinocytes.	[42, 43]
	RB-E2F	P53-p21-RB-E2F	P53 binds to CyclinD, CDK1/2/4, and other cyclins through p21, causing cyclins to be unable to phosphorylate RB. Low phosphorylated RB binds to the E2F promoter, thereby inhibiting cyclin transcription.	[48, 49]

P53 simultaneously regulates multiple downstream pathways, including apoptotic bcl-2-bax, cell cycle regulation such as p16 and RB-E2F, and the dream complex. Elevated p53 expression in diabetes augments apoptotic markers Bax and Caspase-3, which are detrimental to wound healing [10, 45]. Targeted p53 suppression has been shown to enhance the healing process in DW [13, 37, 46]. In injured skin, p53 levels fluctuate over time, inversely correlating with proliferation-associated genes like bcl-2. This p53-bcl-2 dysregulation may impede repair in DW [9].

In DW, p53 overexpression curtails CDK1/2 activity and other proliferative factors, thereby diminishing cellular proliferation [8, 47]. The p53-p21 and RB-E2F pathways are pivotal in cell cycle control[48, 49], with p53-p21 acting as a brake during the G0→G1 and G2→M phase transitions [50], and RB-E2F facilitating the G2-M transition. A suite of genes, including DNA polymerase alpha (POLA1), cell cycle protein A 2 (CCNA2), thymidine kinase 1 (TK1), DHFR, CDK1/2, microchromosome maintenance complex components 3 and 5 (MCM3/5), and DNA replication licensing factors, fall under RB-E2F's regulatory umbrella [51]. The interplay between p53-p21 and the RB pathway, however, remains incompletely understood (Table 1).

### 2.2 The mechanism of p53 inhibiting cell cycle.

**Figure 1.**
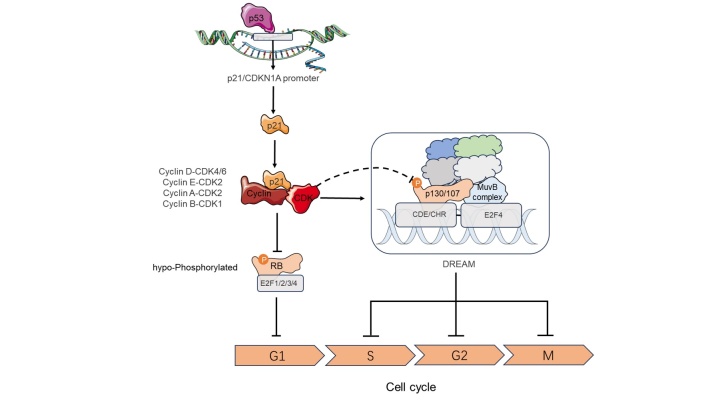
**P53-p21-RB signal transduction**. After activation of p53, the transcription of p21/CDKN1A is strongly induced as a direct target of p53. The cell cycle dependent kinase inhibitor p21 subsequently blocks the activity of several cyclin CDK complexes. This leads to low phosphorylation of RB, which promotes the formation of RB-E2F complexes and their binding to the E2F site in the target promoter. Many target genes are downregulated as a result of this indirect p53 dependent transcriptional inhibition mechanism. Due to the involvement of most suppressed genes in cell cycle progression, their downregulation leads to cell cycle arrest. CDK cyclin-dependent kinase, CDE cell cycle-dependent element, E2F E2 factor, RB Retinoblastoma.

Most cycle genes are regulated by p53 transcription and suppressed [52, 53]. For a long time, it has not been found how p53 down-regulates these cell cycle regulators (such as cyclins B1 and B2) [50]. When we analyzed the whole genome in the context of transcription inhibition dependent on Dream, we found that p53 indirectly inhibited many of its target genes [34]. This transcription inhibition requires CDK inhibitors p21/wa f1/CIP1/ CDKN1a [54-56]. The CDKN1A gene of p21/WAF1/ CIP1/CDKN1A is the first discovered p53 transcription target [57, 58]. P21 can form complexes with CDK1, CDK2, CDK3, CDK4 and CDK6 extensively, and form complexes with these kinases together with specific cyclins[59-62]. The p53-p21 complex is thought to prompt the Dream complex formation, where hypophosphorylated RB binds to E2F4/5, which then associates with cell cycle-dependent element (CDE)/E2F or cell cycle gene homology region (CHR), CDE/CHR promoters [54]. The Dream complex supersedes the Myb-MuvB (MMB) and Forkhead box M1 (FOXM1)-MB complexes, inhibiting G1-S and G2-M phase transitions and down-regulating cell cycle genes [55, 56]. Notably, even with an active p53-p21 pathway, certain cycle-promoting factors may increase, potentially as a countermeasure against senescence [63]. For example, p21 has also been proven to be the assembly factor of Cyclin D and CDK4/6 complex at low concentration [64] (Fig 1). In type 1 diabetic nephropathy, skin fibroblasts exhibit heightened RB phosphorylation, upregulation of cyclin D1, and increased CDK1/CDK4 activity [65]. These insights hint at therapeutic strategies for DW that modulate cell cycle inhibitors like p53 or p21 or enhance cell cycle protein expression to stimulate proliferation [66]. Alternatively, fostering MMB and FOXM1-MMB complex formation could be beneficial [54-56] (Table 1).

P21 has been proven to mediate p53-induced G1 cell cycle arrest[67, 68]. Its coding gene CDKN1A is a prominent p53 target. P53 binds to elements in the p21/CDNKN1A promoter and activates its transcription [69]. However, high p21 levels can also appear independently of p53 [70]. P21 protein inhibits all -CDK pairs involved in the hyperphosphorylation of RB protein, thus forming a complex of hypophosphorylated RB and E2F (preferentially binding to E2F1, E2F2 and E2F3) transcription factors, and binding to E2F binding sites in the target gene promoter to down-regulate transcription [71, 72]. Taken together, these events constitute the p53-p21-RB signaling mechanism, and the formation of the RB-E2F complex depends on the phosphorylation state of RB and is controlled by p21[34]. Several phosphorylation sites carried by RB are substrates of Cyclin D-CDK4/6, Cyclin E-CDK2, Cyclin A-CDK2 and Cyclin B-CDK1 [72-75]. RB-E2F complex inhibits the transcription of many cell cycle genes, many of which are necessary for G1/S conversion [76]. The hypophosphorylation state of RB can be transformed into hyperphosphorylation through the kinase activity of cyclin -CDK complex, these kinases can be inhibited by p21[77]. Therefore, p21 can stimulate the formation of RB-E2F complex, but when p21 loses its function, it can be saved to some extent by CDK4/6 inhibitors such as palbociclib, abemaciclib, ribociclib, and these inhibitors have been used for cancer treatment[78] (Fig. 1).

## 3. P53-dependent chronic skin inflammation in DW.

**Figure 2.**
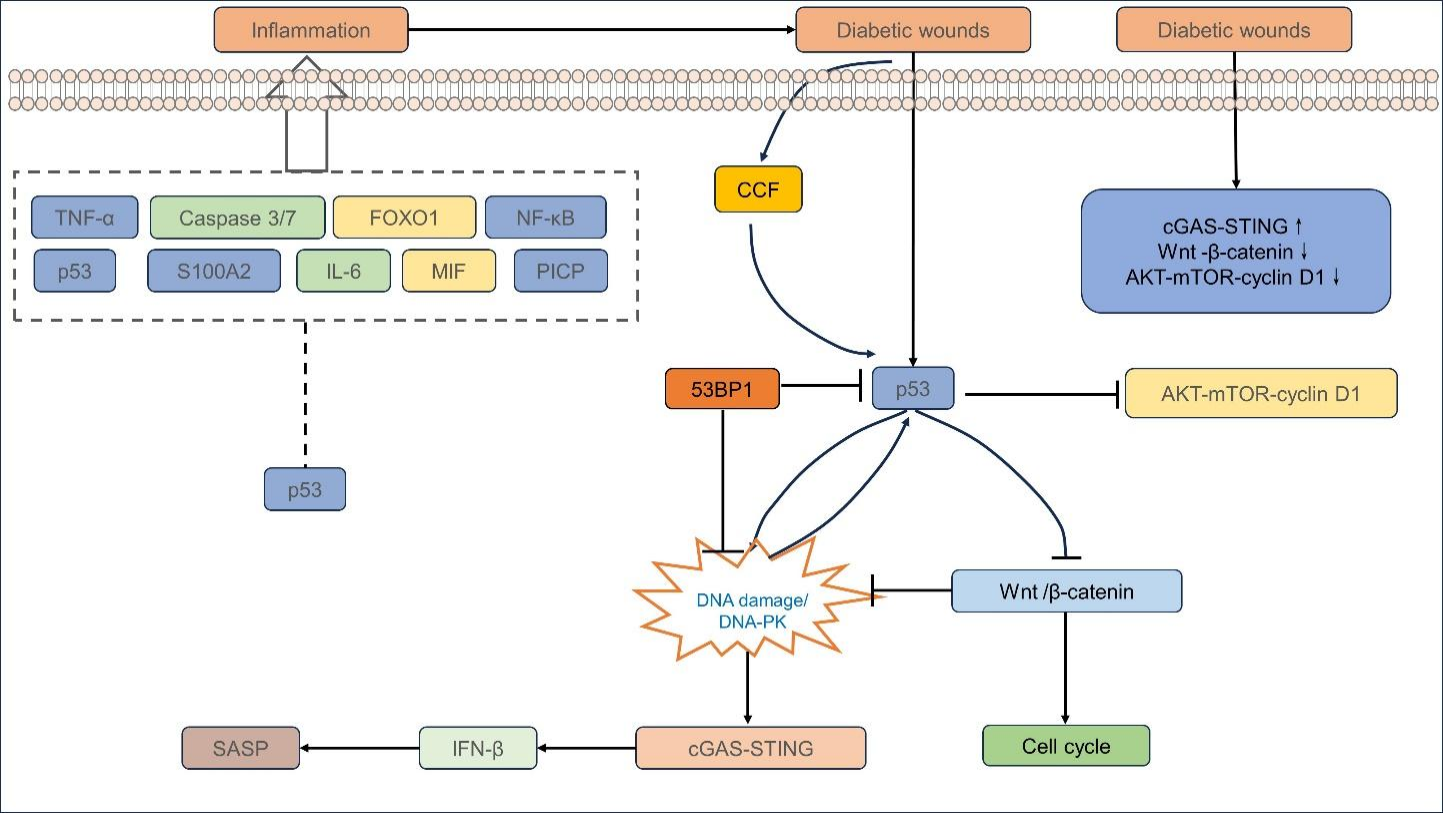
**p53-dependent chronic skin inflammation in diabetic wounds**. In diabetic wounds (DW), keratinocytes, fibroblasts, and macrophages display a highly inflammatory state. Various factors such as TNF-α, p53, FOXO1, S100A2, MIF, Caspase 3/7, IL-6, PICP, and CCF are elevated in these wounds and may be associated with p53. Moreover, abnormal activation of certain inflammatory pathways can be observed in DW, in which p53 likely plays a role. For instance, p53 activation inhibits the AKT-mediated autophagy pathway and the Wnt-β-catenin pathway, while inducing DNA damage to activate the cGAS-STING pathway. Conversely, the inhibition of p53 by 53BP1 can alleviate the inflammatory damage caused by DNA damage. DW diabetic wounds, TNF- α tumor necrosis factor- α, FOXO1 forkhead transcription factor O1, S100A2 formerly called CaN19 or S100L, MIF macrophage migration inhibitory factor, IL-6 Interleukin 6, PICP procollagen C peptide, CCF, AKT protein Kinase B, mTOR mammalian target of rapamycin, cGAS-STING cyclic GMP-AMP- stimulator of interferon genes, SASP senescence-associated secretory phenotypes.

In DFU, a pro-inflammatory state is evident in keratinocytes, fibroblasts, and macrophages [79-81]. Single-cell sequencing of DFU patient skin shows heightened fibroblast inflammation and sluggish migration of monocytes and dendritic cells [82]. Diabetes-induced oxidative stress can activate p53, triggering inflammation [83] (Fig 2).

Numerous investigations have illustrated the potential involvement of p53 in the persistent skin inflammation characteristic of DW [84]. Chronic inflammation is associated with an increase in apoptosis and a reduction in fibroblast proliferation within these wounds. Elevated levels of tumor necrosis factor-alpha (TNF-α), p53, forkhead box O1 (FOXO1), and S100 calcium-binding protein A2 (S100A2) have been reported in the context of DW, with p53 being linked to the regulation of TNF-α and FOXO1[84-87]. It is postulated that the pro-apoptotic transcription factor FOXO1 may act synergistically with TNF-α to upregulate *TP53* and NF-κB expression. Intriguingly, p53 expression is diminished in diabetic skin unless it sustains damage [9]. Furthermore, changes in S100A2 expression can impede keratinocyte migration and proliferation, thereby decelerating the wound healing process. S100A2 is known to potentiate p53-mediated activation of NF-κB, which subsequently inhibits cyclooxygenase-2 (Cox2) and delays cutaneous wound healing. Elucidating the mechanisms by which S100A2 and p53 impede wound healing could unveil new therapeutic avenues for DW [88].

Macrophages are pivotal during the inflammatory phase of DW [89], with macrophage migration inhibitory factor (MIF) playing a regulatory role in inflammation and cellular proliferation, significantly impacting the p53 and E2F-dependent pathways[90]. Exposure of fibroblasts to methylglyoxal, a byproduct of glucose metabolism, results in elevated secretion of interleukin-6 (IL-6) and MIF [91]. The simultaneous expression of type I procollagen C peptide, IL-6, and MIF in primary skin fibroblasts from diabetic individuals suggests that methylglyoxal may impede the healing of diabetic ulcers via MIF-mediated pathways[91]. While MIF has been shown to expedite cutaneous wound healing in murine models [92], the intricacies of its interaction with p53 in DW require further elucidation (Fig. 2).

Additionally, the p53-p21 axis is linked to the DNA damage response in DFU [93]. Activation of p53 can lead to DNA damage, while its deficiency may inhibit such damage [94-96]. The molecule 53BP1 is integral to the response to DNA double-strand breaks, facilitating their repair [97, 98]. Interestingly, p53 can be activated by DNA damage itself [99]. Upon DNA damage or activation by DNA-dependent protein kinase (DNA-PK), p53 can induce cell cycle arrest or apoptosis [100-102]. This indicates that p53 may serve as both a downstream target and a cause of DNA damage. Furthermore, DNA damage can trigger the cyclic GMP-AMP (cGAS)- Interferon genes (STING) pathway, initiating inflammatory responses that may contribute to DW [103-105]. Additionally, mutant p53 is capable of inhibiting cGAS-STING activation under conditions of DNA damage [106].

The aging aspect of diabetes is also noteworthy. In certain senescent states, increased production of cytoplasmic chromatin fragments (CCF) can upregulate p53 expression and activate the cGAS-STING pathway [107, 108]. The downregulation of p53 expression in aging cells can lead to reduced cGAS-STING pathway activation, which may be beneficial for wound healing as it potentially decreases the senescence-associated secretory phenotype (SASP) [109, 110]. However, in the context of diabetic skin ulcers, the interplay between p53 and cGAS-STING appears to be complex. p53 can induce the expression of interferon beta, a component of SASP, which suggests that p53 may contribute to the maintenance of inflammation and senescence in DW. On the other hand, the activation of cGAS-STING by DNA damage, which is common in DW, can further exacerbate inflammation and hinder healing (Fig. 2).

The Wnt-β-catenin signaling pathway is another critical player in the context of DW healing. This pathway is involved in various cellular processes, including cell proliferation and apoptosis, and is regulated by interactions with p53. β-catenin can aggregate with Pygo2 in hair follicle stem cells and keratinocytes, leading to the activation of p53 [111]. While lower expression of p53 might seem beneficial, a p53 deficiency can actually trigger Wnt-β-catenin-dependent systemic inflammation [112], which underscores the importance of a balanced p53 expression for maintaining homeostasis [113].

Moreover, the Wnt-β-catenin signaling pathway can influence DNA damage and cellular senescence. Inhibition of this pathway may promote DNA damage and contribute to the onset of cellular senescence, which is detrimental to wound healing. Interestingly, the Wnt-β-catenin pathway has been shown to play a significant role in DW [114], and therapeutic approaches such as treatment with IL-25 and insulin have been found to restore the down-regulated Wnt-β-catenin signaling in DW, thereby facilitating the healing process [115, 116].

Finally, the increased expression of p53-p21 in fibroblasts from diabetic ulcer patients can lead to the downregulation of the AKT- Mechanistic target of rapamycin (mTOR)-Cyclin D1 pathway. This pathway is critical for cell growth and metabolism, and its downregulation can result in skin aging and delayed wound healing [47] (Fig. 2).

In summary, the intricate interplay between p53 and various signaling pathways, such as cGAS-STING and Wnt-β-catenin, plays a crucial role in the pathophysiology of diabetic skin ulcers. Aberrant activation of inflammatory pathways and the DNA damage response associated with p53 can lead to elevated levels of SASP, which may impede the wound healing process. Understanding these molecular interactions provides insights into potential therapeutic targets for improving wound healing in diabetic patients.

## 4. P53-dependent senescence in DW

**Figure 3.**
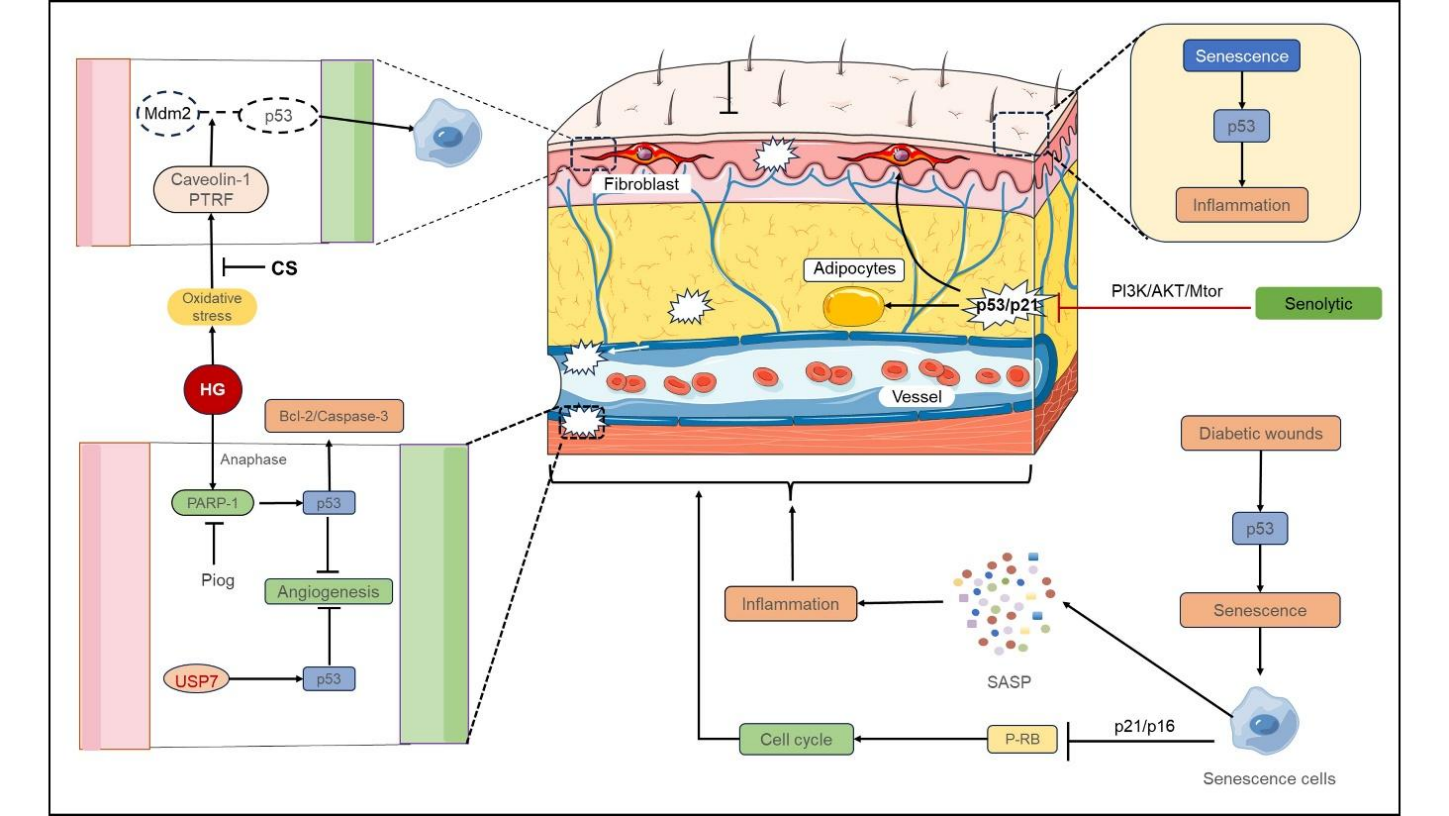
**p53-dependent chronic skin senescence in diabetic wounds**. USP7, as well as HG-induced PARP-1, Caveolin-1, and PTRF, can activate p53, thereby inducing cell ageing and apoptosis. The upregulation of p53 in DW may also be an important factor leading to cell ageing in diabetic skin wounds. Senile cells in DW will release a large amount of SASP, thus inducing the upregulation of inflammatory response and cell cycle arrest. Piog, CS, Senolytic, etc. may inhibit p53 and alleviate the progression of DW. USP7 ubiquitin-specific protease 7, HG high glucose, PARP-1 (ADP)-ribose polymerase-1, PTRF polymerase I and transcript release factor, Piog pioglitazone, CS calcium silicate.

The overexpression of p53 in DFU has been implicated in the induction of cellular senescence and arrest of the cell cycle[117]. This is mediated through the upregulation of the p21 and p16 signaling cascades, leading to the inactivation of RB proteins that are essential for DNA replication, thus precluding the progression of the cell cycle [118]. Recent investigations have elucidated the role of the DREAM complex as a nexus between the p53-p21 and RB-E2F checkpoints, orchestrating the cell cycle dynamics in senescent cells. While the RB-E2F axis is acknowledged as a critical regulator of cellular senescence and proliferation, research specifically addressing the interplay of the p53-p21-RB-DREAM complex-E2F pathway in DW remains absent from the literature (Fig. 3).

Senescent cells are characterized by the secretion of the SASP, which encompasses enzymes such as SA-β-Gal. SASP is composed of a plethora of biologically active cytokines, either directly released by senescent cells or packaged within exosomes [119, 120]. The overproduction of SASP factors can expedite the senescence process [19] and provoke inflammation, leading to localized or systemic tissue damage [121]. Notably, p53-mediated inflammatory responses have been observed in aging skin [122-124], and diabetic skin lesions exhibit an early pro-inflammatory state that may contribute to inflammatory senescence [82, 86, 125].

It is worth noting that p53 is not entirely detrimental to aging. Haploinsufficiency of p53, while overcoming embryonic senescence, does not prevent the onset of premature senescence, including skin wrinkling and delayed wound healing [126] (Fig. 3).

Ubiquitin-specific protease 7 (USP7) has been shown to facilitate p53 ubiquitination, whereas its inhibition promotes p53 deubiquitination, potentially impeding the senescence of vascular endothelial cells in diabetic feet [117]. Additionally, the ROS-dependent p53-Bcl-2-caspase-3 signaling pathway has been implicated in the apoptosis of human umbilical vein endothelial cells (HUVECs) [127], with evidence suggesting a concentration-dependent effect aligned with p53-regulated cell cycle alterations, potentially diminishing angiogenesis in DW [128].

The high-glucose (HG) milieu can induce fibroblast senescence by upregulating caveolin-1 (Cav-1) and polymerase I and transcript release factor (PTRF), leading to the activation of p53 [8]. This Cav-1-PTRF-p53 pathway may also play a role in the delayed healing of DW [8]. Calcium silicate (CS) treatment has been shown to mitigate the HG-induced decline in fibroblast proliferation, migration, and differentiation, correlating with a decrease in ROS production, SA-β-Gal expression, and the expression of p16, p21, and p53 [129] (Fig. 3).

ADP-ribose polymerase-1 (PARP-1), a key player in DNA repair, is also implicated in the regulation of inflammatory cytokines and chemokines, cell proliferation and migration, and oxidative stress responses, thus playing a pivotal role in wound healing. PARP-1 exhibits a dual function in this context; it promotes early endothelial cell repair under high-glucose conditions but can later activate p53 and caspase-3, inhibiting the repair process [130]. Some PARP-1 inhibitors have received FDA approval for use in wound healing therapies [131].

Pioglitazone (Piog) is a medication commonly used in the management of type 2 diabetes, and it works by activating the expression of peroxisome proliferator-activated receptor gamma (PPARγ). This activation can lead to various therapeutic effects, including the improvement of skin-related conditions. For instance, Pioglitazone has been shown to ameliorate dermal atrophy in APOE mice, a model for senescence and age-related diseases [132]. Furthermore, Pioglitazone can reduce the expression of SASP markers, such as p53, superoxide dismutase (SOD), and myeloperoxidase (MPO), indicating its potential role in modulating cellular aging and inflammation [132].

RRAD (Ras-related associated with diabetes) is another molecule of interest that has been identified as both a biomarker and a novel negative regulator of cellular senescence. It appears that RRAD can be influenced by p53 and NF-κB pathways, acting as a negative feedback mechanism to combat senescence. The expression of RRAD is higher in senescent cells, suggesting its potential as a marker for cellular aging [133]. Both p53 and NF-κB are involved in regulating the transcription of RRAD, which can help reduce levels of ROS and counteract senescence [133].

Insulin-like growth factor 1 (IGF-1) is known to be elevated in conditions such as type 2 diabetes and cardiovascular disease, serving as a potential risk marker [134]. In patients with acromegaly, a condition characterized by excessive growth hormone and IGF-1 production, there is an association between reduced life expectancy and an increased incidence of age-related diseases like diabetes. IGF-1 has also been linked to telomere shortening and the upregulation of aging markers such as β-galactosidase, p53, and p21 in human skin fibroblasts [135] (Fig. 3).

Senescent cells, despite their detrimental role in aging and disease, also have beneficial functions. They produce cytokines that aid in tissue repair and prevent excessive fibrosis during wound healing [136-139]. Moreover, they can induce a state of senescence in neighboring cells, potentially converting them into stem cells through the expression of c-Myc (*OSKM*) genes [140].

Senolytic therapy has emerged as a promising approach to target and eliminate senescent cells, which could potentially slow down the aging process and treat age-related diseases[141]. Senolytics are agents that selectively induce apoptosis in senescent cells or inhibit SASP factors [142, 143]. Examples of senolytic agents include dasatinib and quercetin (D+Q), laccase, and extracts from plants like Solidago virgaurea subspecies alpestris, which have shown potential in delaying senescence and improving cell function [144].

Clinical trials for senolytic therapy in diabetic nephropathy are underway, targeting pathways such as phosphoinositide 3-kinase (PI3K)-AKT-mTOR [145]. An extract of the plant Solidago virgaurea subspecies alpestris, which also demonstrated weak senolytic activity, was found to delay the senescence phenotype and improve the function of human dermal fibroblasts [146]. Senolytic exposure was also reported to rescue Zinc Finger Matrin-Type 3 (ZMAT3) methylation, downregulate ZMAT3 expression, improve adipogenesis, and reduce p53 and p21 expression, thus alleviating senescence [142].

Additionally, Silybum marianum flower and indigo leaf extracts also exhibited weak senolytic properties [147, 148]. Other studies have also indicated some plant extracts with low senolytic activity that demonstrated delayed aging and improved function of human dermal fibroblasts [146, 148].

In summary, the research on Pioglitazone, RRAD, IGF-1, and senolytic therapy highlights the complex interplay between diabetes, aging, and cellular senescence. These findings pave the way for the development of novel therapeutic strategies to treat DW and other age-related conditions by targeting the underlying mechanisms of senescence (Fig. 3).

## 5. P53-dependent PANoptosis in DW.

Cellular demise constitutes a multifaceted process encompassing apoptosis, autophagy, necrosis, pyroptosis, ferroptosis, and cuproptosis. Recent investigations have elucidated that such demise is not an isolated event but rather results from intricate interactions among pivotal entities, striking a critical equilibrium between cellular persistence and expiration, and modulating inflammatory signaling. Within this framework, the concept of PANoptosis has been introduced, encapsulating a synergistic mechanism of cell death encompassing inflammasome activation, pyroptosis, apoptosis, and necroptosis. This inflammatory programmed cell death is orchestrated by the PANoptosome complex, which coordinates the aforementioned cell death pathways, including ferroptosis. The nomenclature PANoptosis derives from the constituents of the PANoptosome complex. Significantly, PANoptosis transcends the definition of any singular cell death pathway, such as pyroptosis, apoptosis, or necroptosis [149].

In the clinical milieu, heightened PANoptosis has been observed in patients with severe COVID-19, concomitant with elevated TNF/Interferon (IFN)-γ levels, suggesting an inflammatory demise. Concurrently, upregulation of p21 has been noted, hinting at a link with PANoptosis mechanisms [150]. Although direct evidence connecting p53 to PANoptosis is lacking, current literature points to potential interactions between p53 and cell death processes, specifically ferroptosis and pyroptosis.

**Figure 4.**
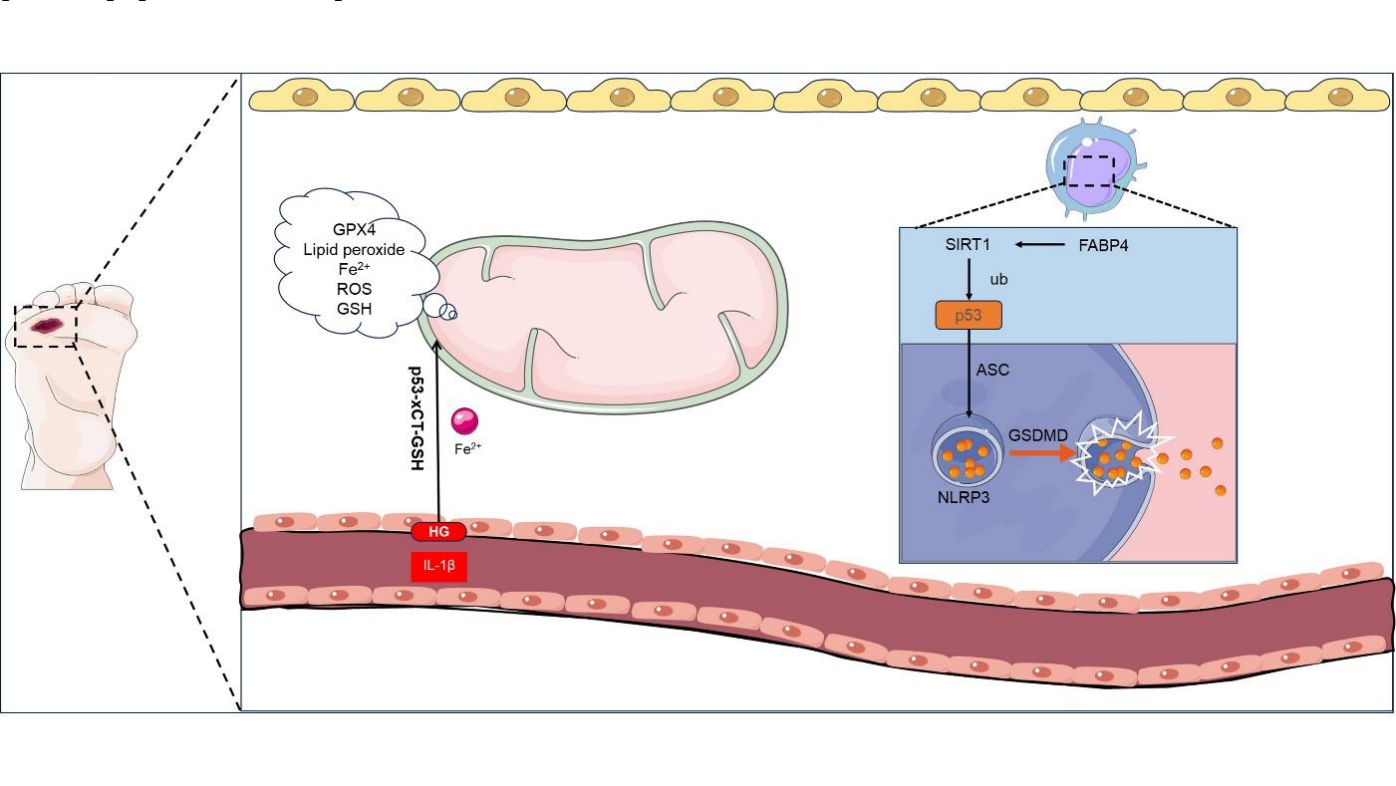
**p53-dependent PANoptosis in diabetic wounds**. The HG environment in DW induces the activation of p53, which in turn activates the xCT-GSH pathway, resulting in increased ferroptosis in vascular endothelial cells. Conversely, the upregulation of FABP4 in macrophages mediates the activation of SIRT1, leading to the ubiquitination activation of p53 and ultimately activating NLRP3-mediated pyroptosis. These factors provide evidence for the existence of PANoptosis in DW and contribute to a slowdown in DW healing. xCT the substrate-specific subunit of system Xc^-^, GSH glutathion, FABP4 fatty acid binding protein 4, SIRT1 sirtuin 1, NLRP3 NOD-like receptor thermal protein domain associated protein 3.

Ferroptosis is an iron-dependent form of cell death, distinguished by ROS accumulation and glutathione depletion, with morphological hallmarks including cellular membrane vesiculation and mitochondrial contraction. It is further characterized by ROS and iron aggregation [151]. Assays for ferroptosis encompass evaluations of ROS, glutathione metabolism, cell viability, lipid peroxidation, divalent iron levels, mitochondrial integrity, and glutathione peroxidase 4 (GPX4) activity [152].

In the context of DFU, ferroptosis expression is elevated [153], and has been demonstrated to mitigate ferroptosis and enhance healing [154]. The *TP53* gene, a central player in DW, exhibits increased expression in diabetic foot tissue compared to healthy tissue [155]. Additionally, in high glucose environments, HG and IL-1β were found to induce desferrioxidation of HUVECs, leading to increased lipid ROS and decreased cell viability [156]. The concurrent activation of the p53-xCT (the substrate-specific subunit of system Xc^-^)-GSH (glutathione) pathway suggests that glucose-induced p53 elevation may trigger ferroptosis, leading to endothelial dysfunction [156]. Additionally, pyroptosis has been identified as aberrantly activated in DW [157].

The apoptosis-associated speck-like protein containing a CARD (ASC or PYCARD) plays a significant role in obesity-related pyroptosis and inflammation. ASC facilitates the assembly of NLRP3 inflammasomes (NOD-like receptor thermal protein domain-associated protein 3) through interactions and recruits procaspase-1 via CARD-CARD interactions, leading to caspase-1 activation and pyroptosis initiation [28, 158, 159]. Acetylation is pivotal in p53 activation, and the suppression of fatty acid binding protein 4 (FABP4) activates SIRT1, which in turn deacetylates p53-STAT, thereby inhibiting pyroptosis and mitigating type 2 diabetes [28]. The interplay between p53 and pyroptosis in macrophages may elucidate the impaired healing observed in DW (Fig. 4).

## 6. The role of p53 in different layers of cells in DW.

A comprehensive analysis of the Gene Expression Omnibus (GEO) database for diabetes-related changes has identified the Mitogen-activated protein kinase (MAPK) pathway as the predominant altered pathway in fibroblasts [160]. Gene differential expression studies have highlighted the roles of *MAPKAPK3* (MAPK-activated protein kinase 3), *HSPA2* (heat-shock protein family A member 2), *TGFBR1* (transforming growth factor-beta receptor 1), and *TP53* in regulating cellular apoptosis, proliferation, differentiation, and inflammation. These findings offer novel insights into the molecular underpinnings of fibroblast dysfunction in DW and may inform the development of targeted therapies [160]. Nonetheless, these postulations necessitate further empirical corroboration.

**Figure 5.**
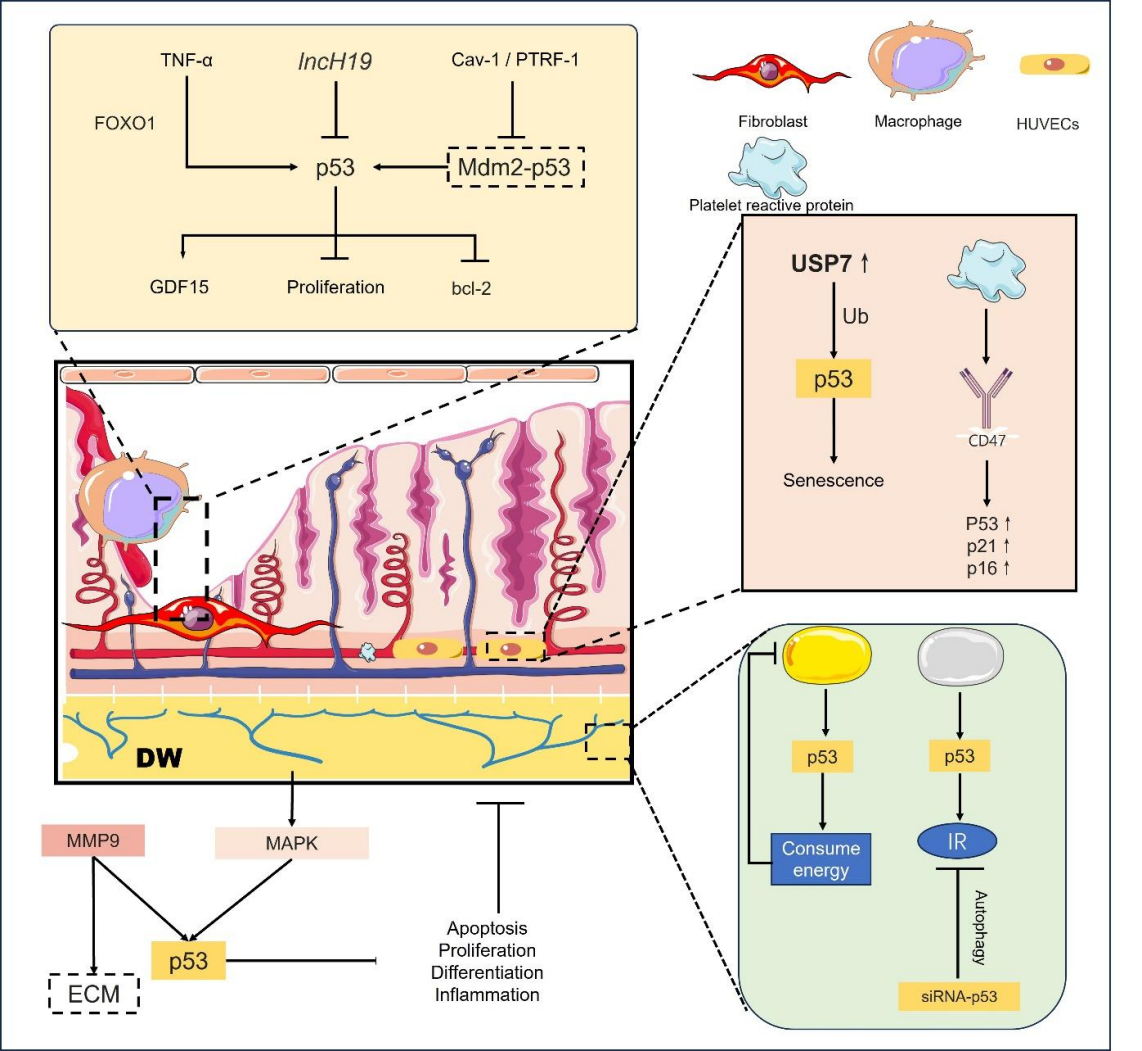
**The role of p53 in different layers of cells in diabetic wounds**. P53 exhibits various roles in different cortical regions of DW. The MAPK and P53 pathways play a part in cell apoptosis, proliferation, differentiation, and inflammation in DW fibroblasts. The absence of lncH19 or the elevation of TNF-α and Cav-1-PTRF-1 could activate p53, which then influences GDF15 and bcl-2, resulting in reduced fibroblast proliferation and impacting macrophage growth. In vascular endothelial cells within the DW, USP7 enhances ubiquitination activation of p53, leading to cellular senescence. Additionally, platelet reactive protein in the DW induces the upregulation of senescent substances, including p53, through CD47. In adipocytes located in the DW, p53 can stimulate energy expenditure in brown adipocytes, thereby inhibiting their growth. Furthermore, p53 can induce insulin resistance in white adipocytes, but transient interference with p53 expression can induce autophagy, consequently down-regulating insulin resistance in white adipocytes. MAPK mitogen-activated protein kinase, Cav-1-PTRF-1 caveolin-1- polymerase I and transcript release factor, GDF15 growth and differentiation factor 15, bcl-2 B-cell lymphoma-2, CD47 Also known as integrin associated protein (IAP), IR insulin resistance.

In macrophages, p53 modulates the activity of GDF15, a key inhibitor of macrophage infiltration and activation [161, 162]. Elevated GDF15 levels, observed in diabetic individuals, have been proposed as a biomarker for type 2 diabetes [163]. The long non-coding RNA H19 (lncH19), a known p53 inhibitor, downregulates GDF15 by suppressing p53 activity. High lncH19 expression in skin fibroblasts has been associated with increased macrophage immune infiltration [11]. Studies indicate that the absence of lncH19 in DW enhances p53 activity and GDF15 secretion, leading to fibroblast cell cycle arrest and a reduction in macrophage presence within affected tissues. Conversely, lncH19 exosome supplementation from adipocytes can attenuate p53 and GDF15 expression, promoting DW healing [11] (Fig. 5).

In the context of DW, the Cav-1-PTRF-1 complex appears to act as an inhibitor of Mouse Double Minute 2 protein (Mdm2)-p53 interaction, consequently suppressing p53 activation and potentially ameliorating oxidative stress while facilitating wound repair [8]. Tumor necrosis factor-alpha (TNF-α) in diabetic skin fibroblasts may modulate p53 levels via FOXO1, culminating in the suppression of fibroblast proliferation [84]. Additionally, keratinocytes in DW demonstrate reduced p53 and bcl-2 expression, without evidence of an antagonistic interaction [93]. This p53 and bcl-2 dysregulation may contribute to the pathogenesis of non-healing DW [9].

Matrix metalloproteinase 9 (MMP9), known for its role in extracellular matrix degradation, exhibits elevated levels in non-healing DW. MMP9 activation can induce cellular apoptosis and inhibit proliferation via upregulation of p53, Fas ligand (FasL), and Fas, thus impeding wound healing [164]. Dermal adipocytes, particularly within dermal white adipose tissue (dWAT), are integral to the wound healing process [165]. p53 is pivotal in modulating both white and brown adipogenesis, inducing senescence and chronic inflammation in white adipose tissue, which exacerbates systemic insulin resistance. Conversely, in brown adipose tissue, p53 activation augments thermogenesis and reduces adipocyte count, underscoring its functional diversity [25, 26, 166]. Transient inhibition of p53 has been shown to obstruct mitochondrial autophagy in adipocytes, which enhances insulin sensitivity in aging white adipose tissue and promotes beige adipocyte recruitment, presenting a potential therapeutic avenue for ameliorating insulin resistance [166].

Ubiquitin-specific protease 7 (USP7) expression is upregulated in DFU and HUVECs are treated with advanced glycation end-products (AGEs). USP7 has been shown to interact with p53, stabilizing it through deubiquitination. Inhibition of USP7 also mitigates AGE-induced cell cycle arrest and senescence in HUVECs, positioning USP7 as a promising therapeutic target for diabetic foot ulcer treatment [117]. Furthermore, diabetes-mediated impairment of angiogenesis and induction of endothelial cell senescence have been linked to upregulated platelet reactive protein-CD47-dependent signaling, accompanied by increased p53, p21, and p16 expression [7] (Fig. 5).

## 7. p53-dependent metabolic pathways in DW.

Mechanistic investigations have elucidated the role of p53 in metabolic reprogramming, predominantly favoring oxidative phosphorylation (OXPHOS) across various cell types. This aligns with the observation that p53 augments glycolysis [167-169]. Through several pathways, p53 enhances OXPHOS, notably by facilitating pyruvate oxidation and augmenting α-ketoglutarate (αKG) concentration within the tricarboxylic acid (TCA) cycle. Furthermore, p53 modulates the pentose phosphate pathway (PPP), essential for the biosynthesis of NADPH and nucleotide precursors imperative for DNA repair [170, 171].

In the realm of lipid metabolism, p53 has a multifaceted role encompassing lipid transport and storage, as well as the biosynthesis of fatty acids and cholesterol, sphingolipid metabolism, and fatty acid oxidation (FAO) [26, 172, 173]. Consequently, p53 is integral to glycolysis, the TCA cycle, OXPHOS, PPP, and lipid metabolism. Within the diabetic milieu, the MDM2-p53-pyruvate carboxylase axis in pancreatic islet β-cells attenuates the production of TCA cycle intermediates, namely oxaloacetate and NADPH, and diminishes oxygen consumption, culminating in glucose intolerance in murine models [174].

Pharmacological inhibition of p53 has demonstrated efficacy in restoring pyruvate carboxylase levels and enhancing β-cell proliferation [174]. A high-fat diet (HFD) precipitates a metabolic shift characterized by reduced glycolysis and a heightened dependence on oxidative fatty acid metabolism. Mice subjected to an HFD display increased p53 expression, which correlates with enhanced palmitate-induced metabolic reprogramming and insulin resistance (IR) via the PANK1-miR-107 axis [175]. Additionally, pyruvate dehydrogenase kinase 4 (PDK4) inversely regulates p53 expression in aging fibroblasts, a factor contributing to DW, and potentially stimulates glycolysis through metabolic reprogramming and ROS [176]. While the regulatory capacity of p53 in metabolic reprogramming is apparent, further exploration is required to delineate its precise mechanisms, particularly in the context of DW.

## 8. P53-dependent drug therapy for DW.

Supplementation with γ-tocopherol has been demonstrated to effectively attenuate p53 expression and inflammatory markers including NF-κB, IL-1β, tumor necrosis factor-α, and C-reactive protein. This modulation enhances wound healing in diabetic murine models [177]. Within the DW milieu, a diverse array of pharmacological agents has been evaluated for their cellular impacts. For example, an active fraction from Piper crocatum Ruiz & Pav (*P. crocatum*) has been identified to suppress p53 expression in hyperglycosylated fibroblasts via the Zn^2+^-binding site, thereby stimulating fibroblast proliferation and upregulating extracellular matrix components such as α-SMA and collagen, potentially expediting wound repair [178].

Calcium silicate (CS), a bioceramic material known for its regenerative capacity, has been observed to downregulate p53 and p21 in hyperglycosylated senescent fibroblasts. It also attenuates reactive oxygen species production and reverses senescence, as evidenced by reduced SA-β-gal expression. Importantly, calcium silicate promotes the differentiation of fibroblasts into myofibroblasts and enhances collagen synthesis, offering therapeutic benefits for DW [129]. Sericata ES has been reported to expedite wound healing in diabetic rats by diminishing p53, MMP-2, and MMP-9 expression in dermal tissue through the modulation of transcription factor AP-1 (JUN) [179].

**Table 2:** Anti-diabetic wounds drugs acting on p53.

Drug	Mechanism	Effect	Ref.
siCav-1, siPTRF	Diabetes-induced oxidative stress upregulates caveolin-1 (Cav-1) and PTRF expression and p53 segregation from Mdm2 in fibroblasts, and depletion of Cav-1 or PTRF inhibits the segregation of Mdm2 from p53, which reduces p53 activation	Inhibiting premature ageing in diabetes.	[8]
PZH	Able to reduce the expression of apoptotic proteins p53, Bax, etc.	Makes wound healing faster.	[12]
Asiaticoside	Asiaticoside works by inhibiting the ROS-dependent p53-Bcl-2-caspase 3 pathway.	Prevents oxidative stress and apoptosis and caspase 3 signal pathway in endothelial cells and accelerates wound healing.	[128]
CS	CS treatment induced down-regulation of p53 and p21 expression in hyperglycosylated senescent fibroblasts, reduced reactive oxygen species production, and improved senescence status (decreased SA-β-gal expression). Moreover, CS also differentiated fibroblasts into myofibroblasts and enhanced collagen deposition.	Accelerating DW healing.	[129]
γ-tocopherol	γ-tocopherol reduced inflammatory factors such as NF-κB, IL-1β, TNF-α and c-reactive protein in DW mice.	Accelerated healing of DW in mice	[177]
*P. crocatum*	The active fraction of *P. crocatum* inhibited the expression of p53 in hyperglycosylated fibroblasts, increased fibroblast proliferation and promoted the expression of extracellular matrices, such as α-SMA and collagen, through the Zn^2+^ binding site.	Accelerating DW healing.	[178]
Sericata ES	Reduction of p53, MMP-2 and MMP-9 expression in dermal tissues by AP-1(c-jun).	Accelerating wound healing in diabetic rats	[179]
PLGA	PLGA nanoparticles significantly shortened the inflammatory period and reduced the expression of p53, p21, bcl-2 and caspase-3.	Accelerating cell proliferation and wound healing.	[180]
hucMSCs	The hucMSCs not only improved cell viability, wound healing, migration and angiogenesis in high glucose-injured HUVEC through a paracrine mode of action, but also significantly reduced the expression of genes such as TNF-α, BAX and p53.	Accelerating DW healing.	[181]
GL	GL significantly reduced the expression of HMGB1 and p53 and significantly inhibited high glucose-induced endothelial cell senescence.	Slowing high glucose-induced vascular endothelial cell senescence.	[182]
Diazoxide	Significantly inhibited the expression of p53 and TSP-1.	Stimulation of angiogenesis in diabetic mice ameliorated impaired BM-EPC function and accelerated wound closure.	[183].
RO	RO also prevented UVB or t-BHP-induced MDF, HUVEC damage and inhibited SA-β-gal, p53 expression.	Preventing cellular ageing.	[186]

Ethanol extracts of propolis-loaded polylactide-co-glycolide (PLGA) nanoparticles have been found to significantly impact wound healing by abbreviating the inflammatory phase and fostering cellular proliferation, thereby hastening tissue repair. These effects are likely due to the modulation of p53, p21, bcl-2, and caspase-3 in fibroblasts [180]. In the context of diabetes-induced oxidative stress, the upregulation of Cav-1 and polymerase I and transcript release factors lead to the dissociation of p53 from Mdm2 in fibroblasts. Gene therapies aiming to deplete Cav-1 or PTRF have shown efficacy in inhibiting the Cav-1-PTRF-p53 pathway, thus preventing premature cellular senescence [8] (Table 2).

Diabetic skin exhibits compromised wound healing and angiogenesis, with heightened p53 expression in vascular endothelial cells. *TP53* gene silencing in wounds using siRNA has been associated with accelerated healing, potentially due to enhanced angiogenic cytokine profiles and endothelial cell markers [13]. Moreover, human mesenchymal stromal/stem cells have demonstrated potential in ameliorating diabetic vascular complications. These cells, via paracrine mechanisms, enhance cell viability, wound closure, migration, and angiogenesis in high-glucose-damaged HUVEC and significantly reduce the expression of pro-inflammatory and apoptosis-associated genes [181].

Glycyrrhiza glabra sweetener has significantly reduced High mobility group B1 (HMGB1) and p53 expression, effectively inhibiting high glucose-induced endothelial cell senescence [182]. The administration of endothelial progenitor cell-derived exosomal miRNA-221-3p has been observed to accelerate skin wound healing in both control and diabetic mice, potentially via the p53 signaling pathway [44]. Diazoxide has been shown to stimulate angiogenesis and improve wound healing in diabetic mice by inhibiting the p53 pathway and promoting the expression of Vascular endothelial growth factor (VEGF), a key factor in blood vessel formation [183].

Asiaticoside, known for its antioxidant properties, not only prevents oxidative stress and apoptosis in endothelial cells by inhibiting the ROS-dependent p53-Bcl-2-caspase-3 signal pathway [128], but also accelerates wound healing [184, 185]. Similarly, pioglitazone/rosiglitazone (RO) has shown to prevent damage to mouse dermal fibroblasts (MDF) and HUVEC caused by UVB or tert-butyl hydroperoxide (t-BHP), inhibiting SA-β-gal and p53 expression, and preventing cellular senescence [128, 132, 186]. Some drugs exhibit promising potential for future DW treatment, although further research is required for validation. Pien-tze-huang (PZH), a traditional Chinese medicine with a long history, has been identified as an effective and convenient treatment for inflammatory diseases such as skin abscesses and ulcers. It has also demonstrated antioxidant effects in DW and reduced the expression of apoptotic proteins such as p53 and Bax, resulting in faster wound healing [12].

In the DFU epidermis, the expression levels of p53, p63, and CD29 are all elevated in keratinocytes, although limited individual studies are available [93]. The use of drugs targeting the p53 pathway has emerged as a promising strategy for treating DW, which are characterized by impaired healing processes. The p53 protein, known for its role in cell cycle regulation and apoptosis, also influences other cellular responses that can impact wound healing. By modulating the activity of p53 and its downstream pathways, these drugs aim to counteract the detrimental effects of diabetic skin senescence and other related complications, such as chronic inflammation and impaired angiogenesis.

In summary, the emerging evidence suggests that targeting the p53 pathway and its associated signaling mechanisms can be beneficial in the treatment of DW. This approach may help to mitigate the cellular senescence, inflammation, and impaired angiogenesis that are characteristic of diabetic skin injuries. However, it is important to note that while the preliminary data is promising, more extensive research and clinical trials are needed to validate the efficacy and safety of these drugs in the context of DW treatment (Table 2).

## 9. Prospects.

The tumor suppressor protein p53 is pivotal in orchestrating cellular processes such as cell cycle regulation, growth facilitation, and maintaining genomic stability during cell proliferation. However, in hyperglycemic conditions, aberrant p53 expression leads to reduced cellular proliferation, enhanced apoptosis, and compromised tissue regeneration. Within the milieu of DW, p53 exerts a profound impact on cellular destiny by modulating metabolic pathways and apoptotic cascades. Its functions extend to arresting the cell cycle and promoting angiogenesis—both of which are integral to the healing process of DWs. The dysregulation of p53-mediated cellular senescence is implicated in the impaired healing observed in DWs. Intriguingly, p53 levels are consistently elevated in patients with diabetes mellitus, irrespective of cutaneous injury.

In the specific context of DWs, heightened p53 activity precipitates cell cycle arrest in essential cellular populations, including fibroblasts and keratinocytes. These cells are crucial for granulation tissue formation and re-epithelialization, processes that are fundamental to wound repair. Certain genes under p53 regulation, as highlighted in this review, emerge as promising therapeutic targets for DW management.

Despite considerable progress in the study of cellular senescence, critical questions linger. Senescent cells display heterogeneity, with differences in characteristics such as telomere dynamics. Moreover, even within a single cell type, the senescence response can vary, complicating the understanding of senescence induction. This heterogeneity is further accentuated when comparing senescent cells across species, particularly in the transition from animal models to human clinical contexts. The diversity among senescent cells presents a challenge in the development of targeted senolytic therapies.

Consensus in the scholarly community points to the need for intensified research in this domain. Exploring pharmacological agents that modulate *TP53* activity, enhance bcl-2 expression, or attenuate the inflammatory response, such as asiaticoside, may offer new avenues for DW treatment. Notably, the activation of p53, especially its acetylated form, is associated with sustained inflammation, which, while initially beneficial for infection prevention, can perpetuate cellular senescence and impede healing. Macrophage polarization is a key factor in this process, with a shift from M1 to M2 phenotypes promoting wound healing by fostering growth factor secretion and reducing inflammatory mediators. The role of glycosylated macrophages in skewing towards a pro-inflammatory polarization, potentially involving p53, merits further investigation. To date, the interplay between p53 and macrophages in DW remains unexplored, representing a significant gap in our understanding of DW pathophysiology that should be prioritized in future studies.

Additionally, phosphorylated p53 may influence DW pathogenesis through various mechanisms, including ferroptosis, pyroptosis, and mitochondrial Bax family-induced apoptosis. Modulating overactive p53 in DW could mitigate the demise of fibroblasts and keratinocytes, thereby enhancing the healing trajectory. Although research is nascent, emerging evidence suggests that p53 may intersect with the nutrient sensing pathway and serve as a regulatory nexus in adipose tissue, with implications for improving insulin resistance (IR) in DM patients. Given that diabetes represents a state of metabolic dysregulation, it is conceivable that it induces alterations in metabolic pathways such as glycolysis and the tricarboxylic acid (TCA) cycle, which are mechanistically linked to p53 activity.

In summary, the prospect of targeting p53 in the treatment of DW offers a compelling avenue for therapeutic intervention. Given its central role in cell cycle regulation, apoptosis, and genomic stability, p53 manipulation could potentially modulate the cellular environment to favor wound healing. In DW, where chronic inflammation and impaired cellular function prevail, normalizing p53 activity may help tilt the balance towards repair and regeneration. Therapeutic strategies could involve the use of small molecules to inhibit excessive p53 activity, thus preventing the apoptosis of key cellular players like fibroblasts and keratinocytes, which are crucial for wound closure. Additionally, understanding the nuanced roles of p53 in metabolic regulation could also lead to treatments that address the underlying metabolic dysfunctions in diabetes, further supporting wound healing. The dual potential to both directly enhances local wound healing and improve systemic metabolic control makes p53 a promising target in the multifaceted approach required for effective DW treatment. However, the complexity of p53's role in cellular processes necessitates a careful and targeted approach to ensure that therapeutic interventions do not inadvertently promote tumorigenesis or interfere with its tumor suppressor functions.
